# Fabrication of Cellulose Nanocrystal/Silver/Alginate Bionanocomposite Films with Enhanced Mechanical and Barrier Properties for Food Packaging Application

**DOI:** 10.3390/nano9111523

**Published:** 2019-10-25

**Authors:** Mithilesh Yadav, Yu-Kuo Liu, Fang-Chyou Chiu

**Affiliations:** 1 Department of Chemical and Materials Engineering, Chang Gung University, Taoyuan 333, Taiwan; ykliu@mail.cgu.edu.tw; 2 Department of General Dentistry, Chang Gung Memorial Hospital, Taoyuan 333, Taiwan

**Keywords:** cellulose nanocrystal, alginate, bionanocomposite films, mechanical property

## Abstract

Eco-friendly cellulose nanocrystal/silver/alginate (CNC/Ag/Alg) bionanocomposite films were successfully prepared by blending of CNC with Ag/Alg solution. The CNC was fabricated from cellulose microcrystal (CMC) by acid hydrolysis method. The Ag nanoparticles (AgNPs) were generated by using Alg as a reducing agent through hydrothermal process. AgNPs-included composite films showed characteristic plasmonic effect of the AgNPs with the maximum absorption at 491 nm and they also showed high ultraviolet (UV) barrier properties. The CNC/Ag/Alg composite films were analyzed by using scanning electron microscopy, transmission electron microscopy, optical microscopy, Fourier transform infrared spectroscopy, and X-ray diffraction technique. Depending on the type of nanofillers, tensile strength of the composite films increased by 39–57% and water vapor permeation decreased by 17–36% compared with those of the neat Alg films. The Ag/Alg and CNC/Ag/Alg films showed brown color as detected from the increase of both ‘b’ and ‘a’ parameters by colorimeter. The UV and water barrier properties of Alg based composite films were found higher than the Alg films. The obtained results suggested that the prepared composite films can be used in food packaging applications.

## 1. Introduction

Plastic packaging films including poly(vinyl chloride), polyethylene, and polypropylene are widely utilized in the market owing to many advantages, such as low price, light weight, good mechanical performance, and water resistance. However, such films cause waste disposal problems in environment due to its non-biodegradable nature [1]. So, today, some of these conventional plastic films are replaced by sustainable-based materials [2,3]. The natural polysaccharides, such as chitosan, alginate (Alg), and carrageenan, etc., are characterized as sustainable materials due to their biodegradability and non-toxicity nature in environment. The films made of such polysaccharides have been studied by researchers for packaging purpose due to their processability and availability [4]. Alg is derived from brown seaweed [5] and consists of a linear block co-polymer of 1,4-linked β-d-mannuronic and α-l-guluronic residues in varying proportions. It is commonly used in food and biotechnology industries because of its biocompatible, low-cost, abundantly available, and environmentally friendly nature. Alg also has great potential in various applications [6,7], which include film forming, gel producing, and emulsion stabilizing features [8,9]. The free standing film of Alg showed poor mechanical and water barrier properties resulted from its hydrophilic feature, thereby limiting the versatility of Alg. Alg was, hence, reinforced by various nanofillers [10] for preparing nanocomposites to overcome its drawbacks in film applications. Silver (Ag) frequently served as a nanofiller in food packaging materials owing to its antibacterial characteristics. Numerous methods were used to synthesize Ag nanoparticles (AgNPs) [11,12], but only few researchers [13,14,15] applied Alg in fabricating AgNPs. The AgNPs were fabricated by Liu et al. [16], who used Alg as a stabilizer under gamma radiation. Narayanan et al. [17] studied the food packaging nature of Ag/Alg nanocomposite films. Tankhiwale et al. [18] and Yoksan et al. [19] reported the successful incorporation of AgNPs in food packaging films as an antimicrobial agent. The food packaging nature of Alg films with others fillers incorporation was also studied by other researchers [20,21,22].

Cellulose is the most abundant natural biopolymer on earth, occurring in wood and cell wall of plants [23,24]. It is composed of β-1,4-linked glucopyranose units that forms a high molecular weight linear homopolymer [25]. Cellulose has many useful applications after its modifications. The chemical and mechanical degradations of cellulose lead to a product of low molecular weight or cellulose microcrystal (CMC). CMC is well-known in applications of food, cosmetic, and medical industries [26]. Cellulose nanocrystal (CNC) can be extracted from plant or it can be extracted from CMC by acid hydrolysis process [27,28]. CNC is an emerging biopolymer with the advantages of abundance, renewability, biodegradability, high mechanical properties, high reinforcing ability, and low density [29]. The geometric dimensions of needle shaped CNC depend on the initial source of cellulose, which accounts for widths of 5–20 nm and lengths of 100 nm–2 μm. The CNC has been reported, playing as nanofiller, to increase the mechanical, and water barrier properties of Alg films for packaging application [30,31]. Dong et al. [32] used CNC/Ag to improve the tensile and antimicrobial properties of chitosan. Yang et al. [33] fabricated AgNPs-bacterial cellulose (BC)/Alg composite by immersing BC/Alg template into AgNO_3_ solution. This composite showed improvement in mechanical, antibacterial, and water swelling ability of pure BC. To the best of our knowledge, the synergistic effects of incorporating CNC and Ag on the mechanical and barrier (water and ultraviolet (UV)) properties of Alg composite film has not been yet reported.

The aim of this study was to fabricate Ag/Alg composite nanoparticles through chemical reduction of Ag^+^ by applying a “green” approach through hydrothermal method of using Alg itself as reductant. Afterwards, the biosynthesized Ag/Alg composite nanoparticles were mixed with CNC to fabricate CNC/Ag/Alg composite films using solution mixing then followed by casting technique. Further, the morphology, interactions among the components, and properties, such as color, mechanical, and barrier (water and UV) of the CNC/Ag/Alg bionanocomposite films were characterized by various techniques.

## 2. Materials and Methods

### 2.1. Chemical and Reagents

Silver nitrate and sodium alginate were obtained from Sigma-Aldrich (Taipei, Taiwan). Sulfuric acid was purchased from J.T. Baker, Taiwan. CMC (Arbocel^®^ UFC100) was received from (J. RETTENMAIER & SOHNE, Saint-Germain-en-Laye, France). Jardino universal potting soil (garden soil) was used for degradation test of fabricated films. Phosphotungstic acid, as staining agent, was received from Sigma-Aldrich. The deionized/millipore water was used in the whole research.

### 2.2. Fabrication of CNC, CNC/Alg, Ag/Alg, and CNC/Ag/Alg Samples

The CNC was fabricated from CMC using sulfuric acid hydrolysis method. The details for CNC fabrication was reported in recently published paper [34]. The obtained CNC was measured with length of 100–500 nm and diameter of 5–30 nm.

The Alg solution was fabricated by dissolving Alg (0.95 g) in 80 mL deionized water with magnetic stirring for 1 h. Water dispersed CNC (by ultrasonication) was charged into the Alg solution to achieve CNC/Alg solution under stirring for 1 h at 27 °C. Afterwards, the CNC/Alg solution was spread on petri dish for film forming. The resulting films were treated with air drying process at 40 °C for 24 h to remove water. The neat Alg film was prepared according to the above mentioned procedure, except without CNC addition.

The Ag/Alg solution was fabricated by mixing of 100 μL of 1 M AgNO_3_ solution and the above Alg solution (1 wt.%). The solution was further put in an oven at 60 °C for 12 h without any stirring. Next, the obtained brown solution of Ag/Alg was kept for magnetic stirring for 12 h. By a similar casting method to the CNC/Alg film preparation, the Ag/Alg composite film was prepared.

**Figure 1:**
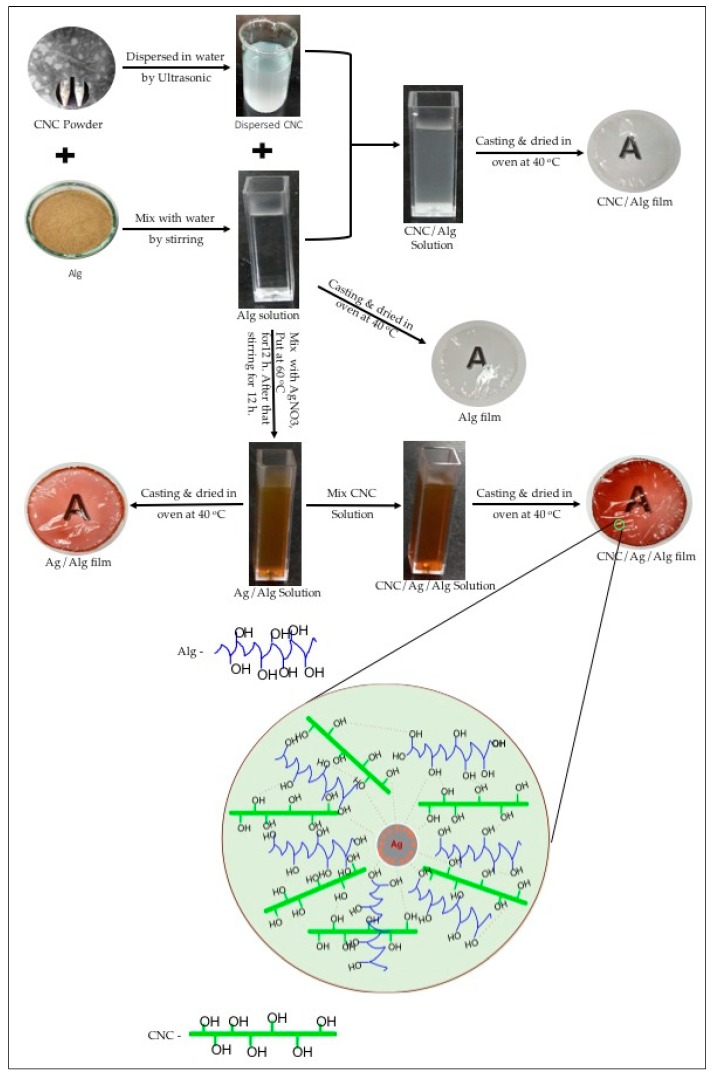
Schematic representation of fabricated solutions and films of alginate (Alg), cellulose nanocrystal (CNC)/Alg, silver (Ag)/Alg, and CNC/Ag/Alg.

The Ag/Alg solution was mixed with CNC solution under stirring for 2 h. The resulting solution was casted on a petri dish to prepare CNC/Ag/Alg composite films. The details about the procedure for fabricating the Alg, CNC/Alg, Ag/Alg, and CNC/Ag/Alg solutions and films are shown in Figure 1. The thickness of all the prepared films was measured with a dial thickness micrometer (SM-112, Dole Teclock, Tongyuan, Japan).

### 2.3. Fourier-Transform Infrared Spectroscopy (FTIR)

The interactions among the components in fabricated composite films were analyzed with FTIR (400–4000 cm^−1^) using Bruker Tensor 27 IR spectrometer (Karlsruhe, Germany).

### 2.4. Field Emission Scanning Electron Microscopy (FESEM)

FESEM (Jeol JSM-7500F, Akishima, Tokyo, Japan) was used for observing the surface and cross-sectional morphology of the prepared composite samples. The Energy-dispersive X-ray spectroscopy(EDS) analysis was carried out to confirm the presence of AgNPs in composite films.

### 2.5. Transmission Electron Microscopy (TEM)

Carbon film covered copper grids (5–6 nm film thickness, 200 mesh, EMS FF200) were used for imaging CNC samples. One drop of the CNC suspension (0.1 mg/mL) was placed on a grid for 30 min and wicked away with a filter paper. Further, the sample was stained by depositing a drop of (2 wt.%) phosphotungstic acid (PTA) solution on the grid for 20 min and wicking the excess solution away with a wet filter paper. The grid was put into oven at 70 °C for drying before insertion into the microscope. Finally, the CNCs were analyzed by a JEOL (JEM-1230 electron microscope, Tokyo, Japan) operated at an accelerating voltage of 200 kV.

### 2.6. Optical Microscopy (OM)

Morphology and dispersion of CNC within (or without) Alg film were examined by OM (Olympus BX-50, Tokyo, Japan).

### 2.7. X-ray Diffraction (XRD)

XRD patterns of all samples were recorded with X-ray diffractometer (D2, Bruker, Karlsruhe, Germany). The crystallinity of CMC and CNC samples was calculated using Equation (1):

(1) $$CI\left(\%\right)=\frac{I_{002}-I_{am}}{I_{002}}\times 100,$$

where I_002_ is the intensity of the crystalline region of cellulose (2*θ* = 22.5°), and I_am_ is the intensity of the amorphous region (2*θ* = 16.3°).

### 2.8. Thermalgravimetric Analysis (TGA)

The degradation behavior and thermal stability of prepared samples were studied in nitrogen atmosphere by operating a thermogravimetric analyzer (TA Q50, TA instrument, New Castle, DE, USA). The samples were heated from 40 °C to 700 °C at a rate of 10 °C min^−1^.

### 2.9. Opacity and UV Visibility

The opacity of prepared films was defined by the following Equation (2) using a UV spectrophotometer (Jasco V-650, Tokyo, Japan) for the measurement:

(2) $$Opacity=\frac{Abs_{600}}{d\;},$$

where Abs_600_ is the absorbance at 600 nm, and d is the film thickness (mm). The transparency of the films was determined following the light transmittance in the light wavelength region (ranged from 190 nm to 900 nm). The used scan rate was 1000 nm/min, and three replicates of each material were measured.

### 2.10. Film Water Solubility (FWS)

The procedure detail of FWS was followed by Yadav et al. [34]. The following, Equation (3), was used to measure FWS of the prepared films.

(3) $$FWS=\frac{W_{i}-W_{f}}{W_{i}}\times 100,$$

where W_i_ and W_f_ represent initial and final dry weight of film.

### 2.11. Moisture Absorption

ASTM D5229 [35] was followed for calculating the moisture absorption of Alg, CNC/Alg, Ag/Alg, and CNC/Ag/Alg films of size 1 × 1 cm^2^ dried at 105 °C for 24 h. The dried films were conditioned in a humid chamber (Giant Force, Xinbei, Taiwan) at 25 °C to attain 95% relative humidity. After a certain time, the films were removed from the chamber and weighed. The following formula [30] was used to determine the moisture absorption (MA).

(4) $$MA\left(\%\right)=\frac{W_{t}-W_{i}}{W_{i}}\times 100,$$

where W_t_ and W_i_ represent the film weight at time t and before exposure to 95% RH, respectively.

### 2.12. Water Vapor Permeation (WVP)

According to ASTM-E96/E96-05, the WVP of prepared films was studied. The details of the procedure was described previously [34]. In each test petri dish, 20 mL of distilled water was added, leaving a distance of approximately 1 cm between the water surface and the film. The film samples were sealed to the dish mouth by a water-resistant sealant. The petri dishes were conditioned in a humid chamber (Giant Force, Taiwan) at 27 °C to ensure 75% RH, and weight was measured at a certain interval of time. Equation (5) was used to calculate the WVP values.

WVP (g m^−1^s^−1^ Pa^−1^) = (w/t). γ. (A)^−1^ (Δp)^−1^, (5)

where w is the weight loss of the petri dishes (g), γ is the film thickness (m), A is the cross-section area of the film (m^2^), t is the time (s), and Δp is the vapor pressure difference.

### 2.13. Color

The color properties of the films were measured by using a TES-135A colorimeter (Taiwan). The obtained parameters are the lightness (L) and chromatic parameters: a (green (–a) to red (+a)) and b (blue (–b) to yellow (+b)) colors. Based on the measured parameters, the total color difference (ΔE) was calculated using Equation (6):

(6) $$\Delta E=\sqrt{{\left(L^{*}-L\right)}^{2}+{\left(a^{*}-a\right)}^{2}+{\left(b^{*}-b\right)}^{2}},$$

where L* = 95.90, a* = −1.16, and b* = +1.56 are the values of standard white plate. L, a, and b are the measured values of the prepared films.

### 2.14. Tensile Properties

The tensile properties of film specimens (10 mm × 60 mm × 0.01 mm) were determined (according to ASTM D638) using a Gotech AI-3000 system (Taichung, Taiwan). A crosshead speed of 10 mm/min and, initial grip distance of 40 mm were used for the tests. The pull tests of the films in terms of tensile strength and elongation at break (%) was calculated by following formula:

(7) $$\mathrm{Tensile}\;\mathrm{strength}\;\left(\frac{N}{{\mathrm{mm}}^{2}}\right)=\frac{\mathrm{Breaking}\;\mathrm{force}\;\left(N\right)}{\mathrm{Cross}\;\sec\mathrm{tional}\;\mathrm{area}\;\mathrm{of}\;\mathrm{the}\;\mathrm{sample}\;\left({\mathrm{mm}}^{2}\right)}\times 100,$$

(8) $$\mathrm{Elongation}\;\mathrm{at}\;\mathrm{break}\left(\%\right)=\frac{\mathrm{The}\;\mathrm{increase}\;\mathrm{in}\;\mathrm{length}\;\mathrm{at}\;\mathrm{breaking}\;\mathrm{point}\left(\mathrm{mm}\right)}{\mathrm{Original}\;\mathrm{length}\left(\mathrm{mm}\right)}\times 100.$$

### 2.15. Biodegradability

Soil burial tests for biodegradation of the films were conducted by a method described by Martucci et al. [36]. The films were cut into rectangular pieces (2 cm × 2 cm), dried in an oven at 105 °C for 6 h, and weighed (W_i_). A total of 35 g of soil was poured into a plastic pot up to a thickness of approximately 1 cm. Then, the films were buried under soil at a depth of 0.5 cm from the soil surface. The assay was completed at 25 °C and (30%) RH by using a humid chamber (Giant Force, Taiwan). Films were taken from the soil at different times and cleaned carefully with tissue paper. Consequently, the films were dried in an oven at 105 °C for 6 h and weighed (W_t_) to determine the percentage of weight loss using the following equation:

(9) $$Weight\;loss\;\left(\%\right)=\frac{W_{i}-W_{t}}{W_{i}}\times 100.$$

## 3. Results

### 3.1. Fourier-Transform Infrared Spectroscopy

**Figure 2:**
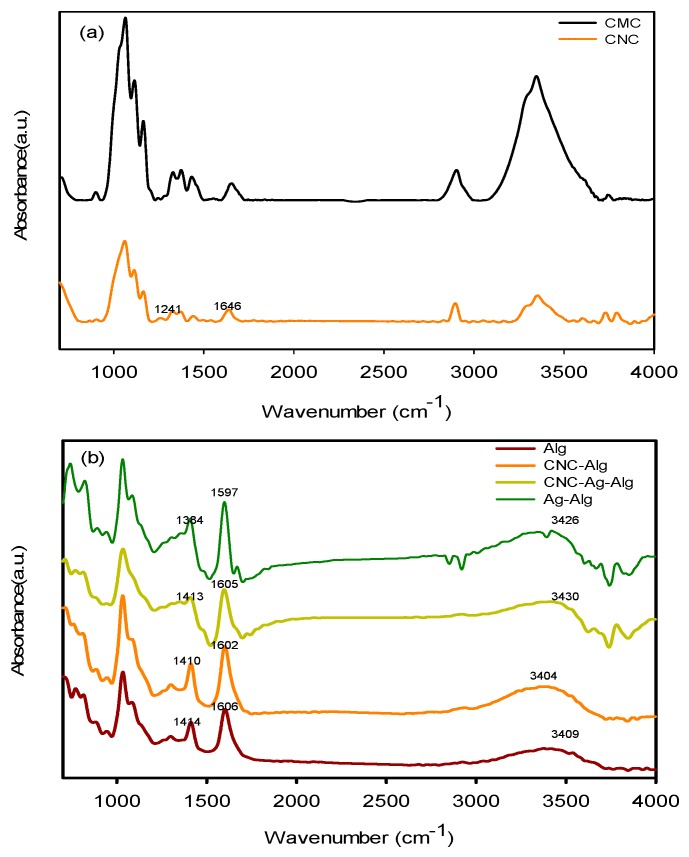
FTIR spectra of (**a**) cellulose microcrystal (CMC) and CNC (**b**) Alg, CNC/Alg, CNC/Ag/Alg, and Ag/Alg samples.

FTIR spectra of CMC, CNC, Alg, Ag/Alg, CNC/Alg, and CNC/Ag/Alg samples are shown in Figure 2a,b. The existence of two new peaks at 1646 and 1241 cm^−1^ in CNC spectrum confirmed that CMC was acidified. In the Alg spectrum, the characteristic peaks at 1606 (asymmetric stretching vibrations of carboxylate anions), 1414 (symmetric stretching vibrations of carboxylate anions), and 3409 cm^−1^ (O–H stretching vibration) [37] were assigned. When AgNO_3_ was added to Alg, the peaks at 1606–1597 and 1414–1384 sharpened, thereby suggesting that AgNPs were generated by the reduction of Ag^+^ with Alg [13]. The shifting of peak from 3409 cm^−1^ (in Alg) to 3426 cm^−1^ (in Ag/Alg film) suggests a certain degree of interaction occurred between Ag and O. The functional groups (–OH and –COOH) of Alg should be involved for fabricating AgNPs [38]. After CNC was added to Alg, the peaks at 3409–3404 (O–H stretching vibration), 1606–1602, and 1413–1410 cm^−1^ sharpened, thereby proving the miscibility (due to presence of hydrogen bonding) [39] between CNC and Alg. When CNC was added to Ag/Alg, the peaks at 3426–3430 (O–H stretching vibration) and 1597–1605 and 1384–1413 cm^−1^ sharpened, confirming again that hydrogen bonding type interaction occurred between Ag/Alg and CNC.

### 3.2. Morphology

**Figure 3:**
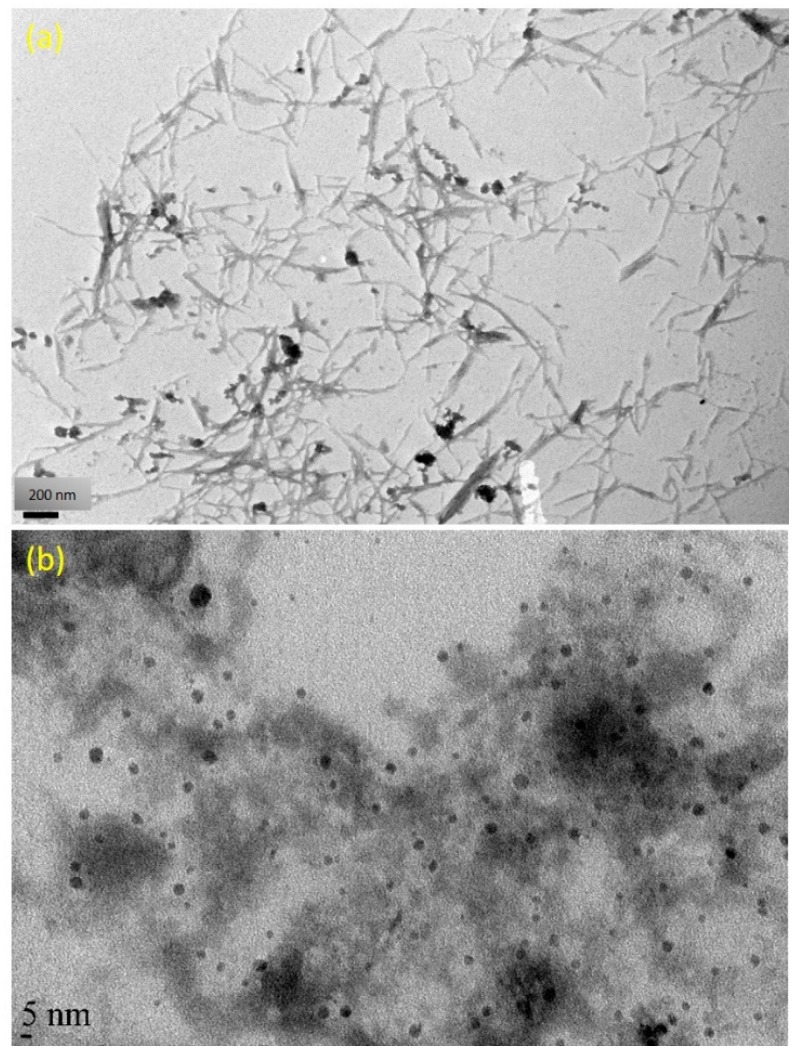
TEM images of (**a**) CNC and (**b**) AgNPs in Ag/Alg samples.

**Figure 4:**
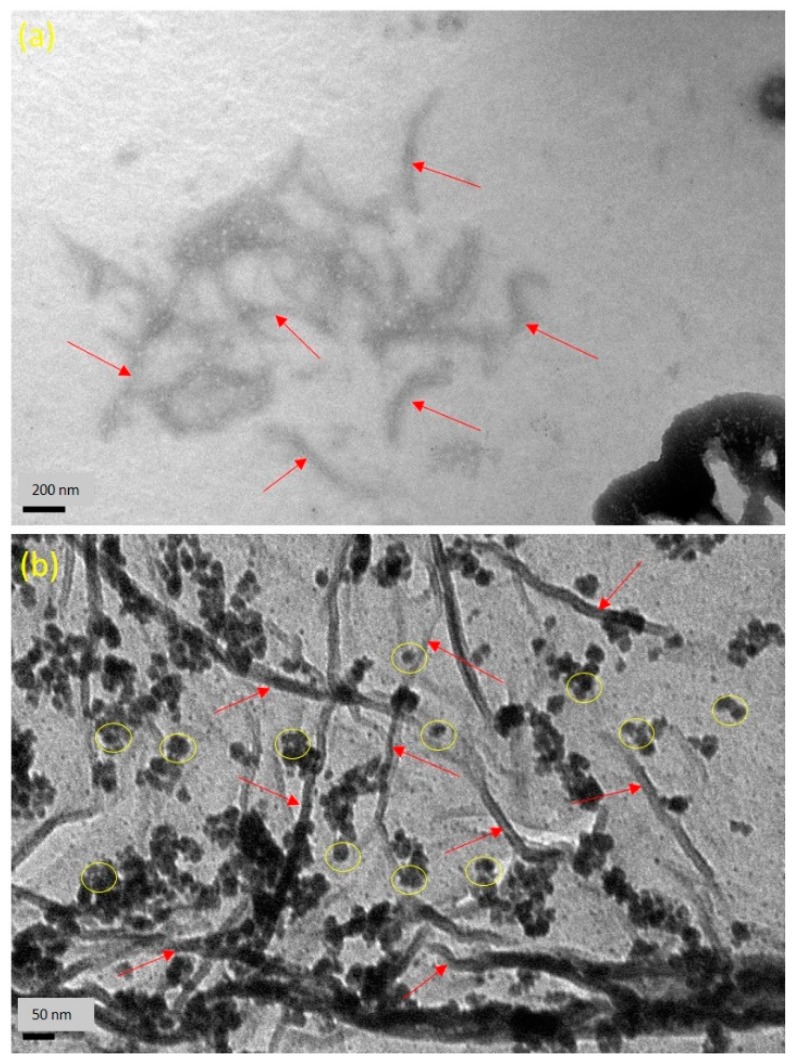
Transmission Electron Microscopy (TEM) images of (**a**) CNC/Alg and (**b**) CNC/Ag/Alg composite films.

The TEM images of fabricated CNC and AgNPs are presented in Figure 3a,b. As seen from Figure 3a, it confirmed that the lengths and widths of the needle shaped CNC were found 100–500 and 5–30 nm, respectively. The averaged particle size of AgNPs was in the range of 4.8 nm, as revealed in Figure 3b. A typical TEM image of Ag/Alg film, as presented in Figure 3b, manifests that spherical AgNPs (the small black dots) are finely dispersed within Alg fibers [40]. When AgNO_3_ was immersed in Alg solution, Ag^+^ could easily diffuse into Alg fibers and reacted with the fibers via ion exchange with sodium ion in the fibers due to the negatively charged Alg facilitating the attraction of the positively charged Ag ions by electrostatic attraction [41]. Then, number of Ag^+^ ions decreased in situ, and the formed AgNPs closely deposited in the Alg fibers. Moreover, fine dispersion was observed, indicating that the in situ formed AgNPs could be effectively stabilized by the Alg fibers. Figure 4a shows the dispersed needle-shaped CNC in CNC/Alg film, confirming the mixing of CNC in the Alg matrix [42]. The TEM image of CNC/Ag/Alg composite, shown in Figure 4b, confirmed fine dispersion of nanofillers (Ag and CNC) in the samples, specifying that Alg acted as reducing agents. Similar results have been previously reported [43]. The arrows in Figure 4a,b indicate the dispersed CNC. The cross-sectional morphology of CNC/Ag/Alg composite film, as observed by FESEM in Figure S1a,b, indicate the existence of spherical AgNPs. EDS analysis, shown in Figure S1c,d, confirms the existence C, O, Ag, and Na elements in the composite film. The OM images of CMC and CNC in dry powder form, and Alg, Ag/Alg, CNC/Alg, and CNC/Ag/Alg composites in film form are presented in Figure S2. It is noted that Ag and CNC were well-dispersed in the Alg matrix and might lead to improved barrier and mechanical performance.

### 3.3. X-ray Diffraction (XRD)

**Figure 5:**
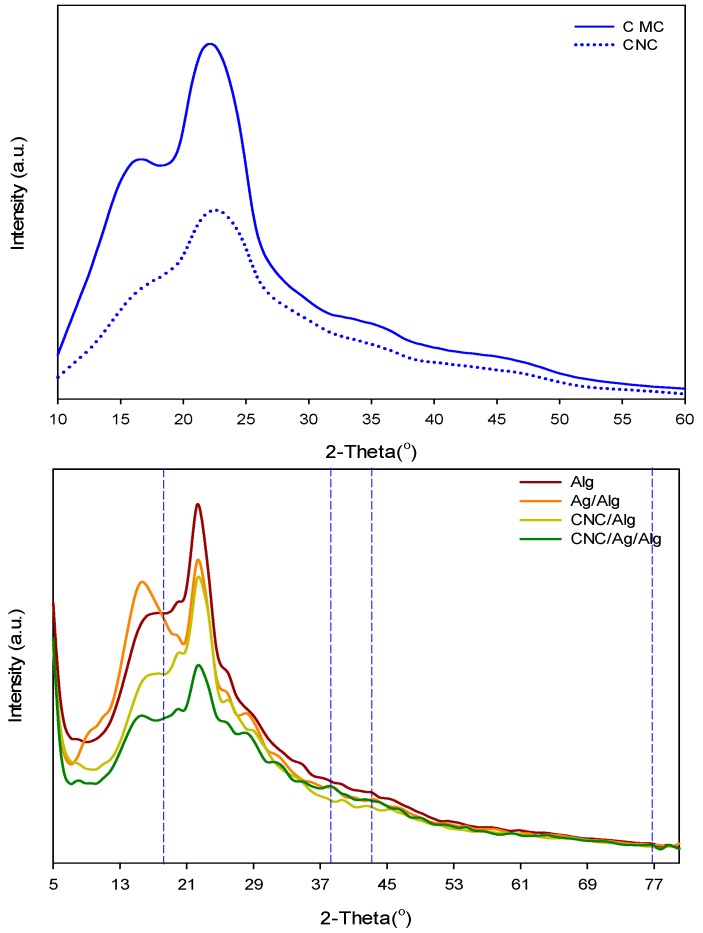
XRD patterns of CMC, CNC, Alg, Ag/Alg, CNC/Alg, and CNC/Ag/Alg samples.

Figure 5 exhibits XRD data (2θ = 5° to 80°) of CMC, CNC, Alg, Ag/Alg, CNC/Alg, and CNC/Ag/Alg samples. As shown in the Figure 5, CMC showed four crystalline peaks at 22.6°, 15.3°, 16.5°, and 34.7°. However, CNC presented crystalline peaks at 23.0°, 15.6°, 16.4°, and 34.6°, indicating a crystal structure of cellulose I [44]. On the basis of obtained crystallinity indexes (CMC = 0.50, CNC = 0.58), it is noticed that CNC is more crystalline than CMC. The two broad peaks of Alg at 16.63° and 22.35 show its amorphous nature. The Ag/Alg composite films exhibited three XRD peaks at 38.27° (111), 43.62° (200), and 76.72° (311), which confirmed the existence of Ag in bionanocomposite film [45]. After CNC loading with Alg, the peak 22.9° of CNC disappeared [46,47]. As shown in Figure 5, incorporation of 5 wt.% CNC decreased the intensity of Alg peaks. The similarity between the XRD curve of CNC/Alg and CNC suggests that CNC are well-distributed in polymer solution. The XRD pattern of CNC/Ag/Alg bionanocomposite films is almost the same as that of Ag/Alg, thereby confirming that Ag is present in the blends.

### 3.4. Thermal Properties

**Figure 6:**
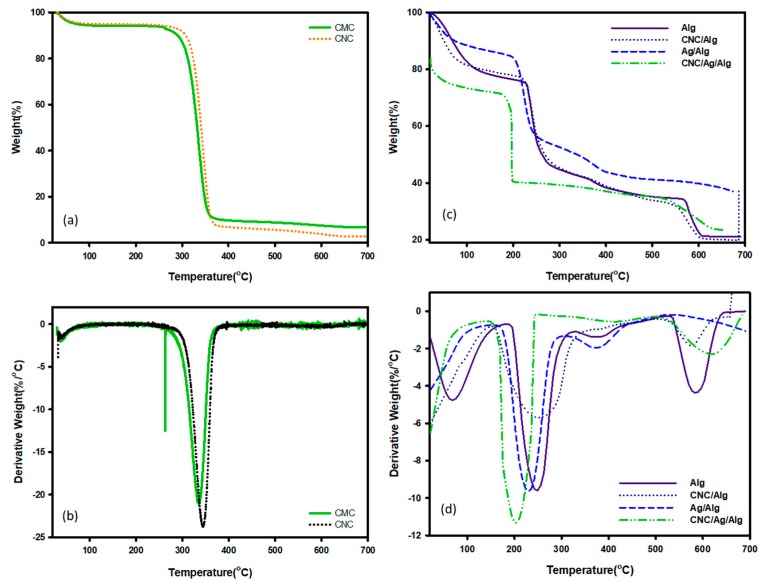
TGA (**a**,**c**) and DTGA (**b**,**d**) curve of CMC, CNC, Alg, CNC/Alg, Ag/Alg, and CNC/Ag/Alg samples.

Biomaterials based packaging films are broadly used in the food industry because of their high yield, less handling time, and less production cost, but temperatures lower their stability in terms of degradation. Recently, a paper published by Otoni et al. [48] studied in details for the thermal properties of fruits and vegetable-based food packaging films. The thermal stability of these films can be understood with TGA in terms of integral procedural decomposition temperature (IPDT) values. Proposed by Doyle et al. [49], IPDT is associated with the extent of volatility of polymeric materials and has been used to estimate the inherent thermal stability of polymeric materials. IPDT is calculated from Equation (11):

(10) $$IPDT\left({}^{\circ}C\right)=A^{*}K^{*}\left(T_{f}-T_{i}\right)+T_{i},$$

(11) $$A^{*}=\frac{S_{1}+S_{2}}{S_{1}+S_{2}+S_{3}},$$

(12) $$K^{*}=\frac{S_{1}+S_{2}}{S_{1}},$$

where, A* is the area ratio of the total experimental curve defined by the total TGA thermograms. T_i_ and T_f_ are the initial and final experimental temperature, respectively. A presentation of S_1_, S_2_, and S_3_ for calculating A* and K* are provided in a published research paper [50]. Figure 6a,b pointed to the TGA/ Differential Thermal Gravimetric Analysis (DTGA) curve of CMC and CNC. As found in the figure, CMC and CNC showed almost the same thermal degradation behavior. The degradation at 346 °C was found due to the burning of cellulose, dehydration, and decomposition of the glycoside units [51]. The IPDT values (presented in Table S1) indicated that CMC is more thermally stable than CNC. Figure 6c,d showed the TGA/DTGA curve of Alg and its nanocomposite samples. The thermal degradation of Alg film was previously reported by Oun and Rhim et al. [52]. The degradation at 204–315 °C might be due to the vaporization of glycerol and polymer matrix Alg [52]. A further temperature increase to 700 °C showed third and fourth peaks at approximately 374 (347–417) °C and 575 (541–627) °C, respectively. The TGA/DTGA of Ag/Alg bionanocomposite films showed three-step degradation. Ag/Alg bionanocomposite films variously degraded at 40–112, 191–301, and 346–418 °C, with T_max_ values of 54, 238, and 393 °C identified in the three-step degradation (DTGA, Figure 6d). The degradation showed in TGA/DTGA of (5 wt.%) CNC/Alg bionanocomposite films occurred in three steps. Specifically, (5 wt.%) CNC/Alg degraded at 31–105, 205–322, and 527–625 °C, with Tmax values of 48, 247, and 572 °C identified in the three-step degradation (DTGA, Figure 6d). From obtained results, CNC/Alg films showed higher thermal stability comparison to neat polymer matrix and CNC. This behavior is also verified by IPDT (Table S1) values [31]. As shown in the figure, CNC content affected the phase of used Alg films. The first (32–124 °C) and second (124–242 °C) degradation occurred due to water desorption and fiber dehydration [53], respectively. At the end, carbonaceous type materials were removed at 242 °C [54]. The degradation of CNC/Ag/Alg bionanocomposite films in TGA/DTGA occurred in two steps rather than one. Specially, CNC/Ag/Alg bionanocomposite films degraded at 178–249 °C and 530–677 °C, with T_max_ values of 232 and 622 °C recognized in the two-step degradation (DTGA, Figure 6d). Furthermore, IPDT values of Alg, Ag/Alg, CNC/Alg, and CNC/Ag/Alg films obtained on the basis of Doyle concept are shown in Table S1. The order of Alg, Ag/Alg, CNC/Alg, and CNC/Ag/Alg films for IPDT can be represented as

Alg < CNC/Alg < Ag/Alg > CNC/Ag/Alg

601.934 < 642.551 < 1078.890 > 766.312.

A comparison of the thermograms of Alg, Ag/Alg, CNC/Alg, and CNC/Ag/Alg films confirmed that the IPDT values were highest for Ag/Alg films, thereby indicating that Ag/Alg composite films is more thermally stable than Alg, CNC/Alg, and CNC/Ag/Alg films.

### 3.5. Water Barrier Properties of the Films

Lower water solubility of food packaging films is an important property for applications in food protection because water resistance avoided films disintegration during coating of humid food surfaces. The FWS of all used films were presented in Table 1. As shown in the table, Ag/Alg, CNC/Alg, and CNC/Ag/Alg bionanocomposite films exhibited FWS value at 89.39, 61.29, and 56.36 which is lower than neat Alg film (99.20). The low solubility data may be caused by high crystallinity of CNC and their strong hydrogen-bonded network within the Alg matrix [55]. This low solubility of samples can be attributed to the presence of nanoparticles [56,57].

The moisture absorption property of biopolymer food packaging films depends on water sensitivity and hygroscopic properties. Sometimes, these properties restricted the application of Alg film. In this regard, Alg was reinforced with Ag and CNC. So, the chances to absorb moisture were decreased due to interaction of Alg, Ag, and CNC. As illustrated in Table 1, the MA value of the Ag/Alg bionanocomposite films decreased from 18.87% to 12.33% because of the presence of lesser number of –OH groups of the Alg matrix that contributed in bonding with Ag. The provided data in Table 1 confirmed that the MA of neat Alg films decreased from 18.87% to 12.22% after loading of (5 wt.%) CNC. It might be due to the availability of lesser number of –OH group for hydrogen bonding with CNC (3-D network hard materials). In the case of CNC/Ag/Alg composite films, a decrement of MA was also recorded from 18.87% to 13.24% compared with neat Alg.

Water vapor permeation (WVP) provides an information about the water transfer from the food to its environment. The WVP of Alg, Ag/Alg, CNC/Alg, and CNC/Ag/Alg are presented in Table 1. From the table, it is clear that the WVP of CNC/Alg bionanocomposite films was higher than that of Ag/Alg bionanocomposite films, and the WVP of CNC/Ag/Alg bionanocomposite films was found in between both. It might be due to the presence of CNC and AgNPs structure. The needle shaped CNC distributed in the Alg might improve an effective tortuous route [31] for vapor diffusion process than AgNPs. Published research papers [11,34] also described that the water barrier behaviors were improved if the nanofillers are less permeable than polymer matrix.

### 3.6. Thickness and Mechanical Properties of Films

The thickness of packaging films generally depend on the three factors [58]: structure, composition, and interaction of the solutions. Meanwhile, the thickness of the synthesized films (Table 1) varied from 10 μm to 12 μm. Among the films, neat Alg film exhibited least thickness (10 μm). However, CNC reinforced Alg films (CNC/Alg) enhanced the thickness to 11 μm. The prepared CNC/Ag/Alg bionanocomposite films showed a maximum thickness of 12 μm. The mechanical behavior of packaging films is a highly important property for the film to maintain its authenticity and bear environmental stress during packaging application [59]. This behavior is generally studied in terms of Tensile Strength (TS) (strength) and Elongation at break(EB) (flexibility). These features support the correlation of the mechanical properties of films to their compositions and chemical structures. The TS and EB (%) of the Alg films were valued at 25.60 MPa and 17.10%, respectively, thereby indicating that the Alg film is mechanically stronger than PLA films [60]. However, the flexibility of the Alg film is particularly low. From Table 1, it is revealed that the TS of Ag/Alg, CNC/Ag/Alg, and CNC/Alg bionanocomposite films was found 57.42, 53.91, and 38.67%, respectively, higher than Alg film. The TS of used films are comparable with LDPE (8–10 MPa), HDPE (19–31 MPa), EVOH (6–19 MPa), PCL (4 MPa), PS (31–49 MPa), PLA (45 MPa), PVC (42–55 MPa), PP (27–98 MPa), and PET (157–177 MPa) plastic films. Huq et al. [31] reported that 5 wt.% CNC loading significantly increased the mechanical and barrier properties of the Alg-based matrix. Deepa et al. [30] found that the incorporation of 10 wt.% CNF into the Alg matrix improved the mechanical properties. Shankar et al. [40] observed that Ag/Alg bionanocomposite films exhibited greater TS (63.2 MPa) than the Alg (56.8 MPa). A similar improvement in TS of Alg by loading Ag, CNC, clay, and graphene oxide were also reported in numerous papers [30,31,40,61,62]. The increase in mechanical strength of the nanocomposite films is mainly attributed to the physical attraction between the filler and the polymer matrix [63,64]. Huq et al. [31] reported that the high TS of the Alg-based bionanocomposite films is due to good interfacial interaction between the nanofillers and Alg-based matrix because of similar polysaccharide structures of cellulose and Alg. The other explanation for the improvement in TS of the Alg-based bionanocomposite films may be the increase of contact area between Alg and nanoparticles [63]. Some other research groups [65] noticed that AgNPs based biocomposites were performed by interactions between the hydroxyl groups of biopolymer and the partial positive charge on the surface of the AgNPs. On the other hand, EB (%) of CNC/Ag/Alg, CNC/Alg, and Ag/Alg decreased by 14.70%, 14.30%, and 13.20%, respectively (Table 1). The obtained EB (%) of composite films is comparable with PS (2–3%), PVC (20–180%), HDPE (20–50%) [66], and PVDC (10–40%) films. A similar trend in EB was also observed by Rhim et al. [63] and Mathew et al. [64]. They explained that the decrease in EB of bionanocomposite films is due to decreased ductility of the polymer by using nanoparticles. The tensile strength of CNC/Alg, Ag/Alg, and CNC/Ag/Alg bionanocomposite films maintained a tensile nature better than the Alg. Thus, the used nanofillers, such as Ag, CNC, and Ag/CNC, can be used as a reinforcing agents in the Alg films.

### 3.7. Color Analysis

**Figure 7:**
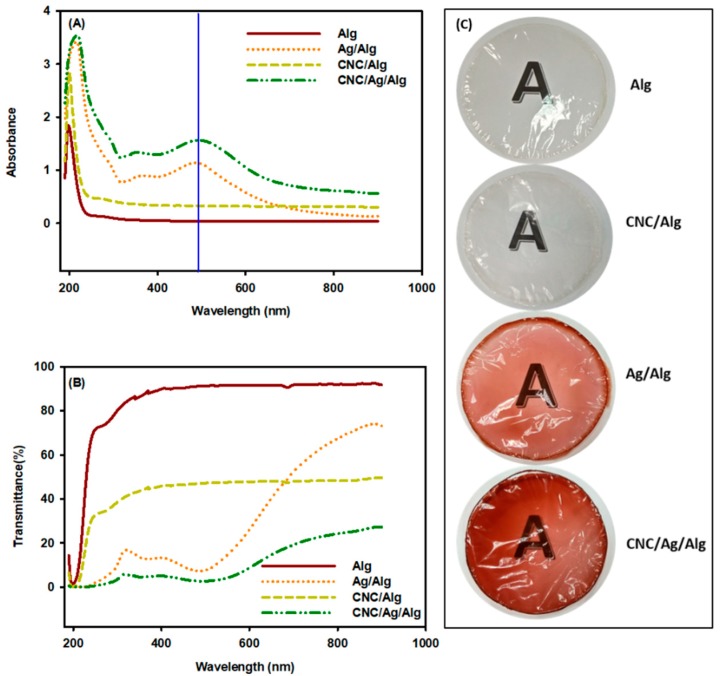
Ultraviolet (UV) spectra for (**A**) absorbance, (**B**) transmittance, and (**C**) visual observation (transparency) of biocomposite films.

The physical appearance of the food product affects the consumer preferences. Therefore, the color of the packaging films directed a great effect in food industry. The obtained color variables L, a, b, and ΔE are presented in Table 2. As seen from Table 2, Ag/Alg and CNC/Ag/Alg composite films showed a darker color than Alg as revealed from the decreased values of L. The Ag/Alg and CNC/Ag/Alg films showed brown color as detected from the increase of both ‘b’ and ‘a’. The value of total color difference (ΔE) of AgNPs incorporated composite films (Ag/Alg and CNC/Ag/Alg) increased dramatically. This is might be due to the development of brown color caused by the plasmonic effect of AgNPs [67]. The plasmonic effect of AgNPs was presented in the absorption spectra of the films, as displayed in Figure 7.

### 3.8. Opacity and UV Visibility of Films

The ability of the food packaging films to block the UV light is an important attribute for food products. Thus, the transmission of ultraviolet (200–400 nm) and visible light (400–700 nm) through food packaging films played an important role for tracing proper package. The biopolymer based transparent food packaging films secure the pictorial confirmation of food content, which is an important reason that affects the consumer’s sense of food quality. Opacity is a key factor that controls the quality of packaging films. As presented in Table 2, the opacity of all used films showed the following order:

Alg > CNC/Alg > Ag/Alg > CNC/Ag/Alg

1.37 > 11.43 > 20.84 > 37.70

The optical properties of Alg, Ag/Alg, CNC/Alg, and CNC/Ag/Alg observed by UV−Vis absorption spectrophotometry are shown in Figure 7. No absorption band was found in the 300−650 nm region for Alg and CNC/Alg (Figure 7A). By contrast, an absorption band at 491 nm was observed in Ag/Alg and CNC/Ag/Alg (Figure 7A). Fundamentally, AgNPs exhibited a surface plasmon resonance (SPR) band between 350 and 500 nm [68]. The SPR band of Ag/Alg and CNC/Ag/Alg is attributed to the presence of AgNPs. Ag/Alg showed no band shift compared with CNC/Ag/Alg due to surface plasmon resonance. This occurrence implied AgNPs are well-dispersed within the Alg matrix; otherwise, band shift occurs due to AgNPs aggregation [69,70]. Evidently, the well dispersion of AgNPs in Ag/Alg and CNC/Ag/Alg was presented in TEM images Figure 4a,c. Figure 7B illustrates that the UV light transmittance of the Alg nanocomposite films continuously decreases compared with that of Alg films due to the UV-shielding ability of Ag, CNC, and CNC/Ag. The decline in transmittance with Ag, CNC, and CNC/Ag contents is symbolic of the uniform distribution of Ag, CNC, and CNC/Ag in the Alg matrix [34]. As shown in the figure, the UV light transmittances of Alg, Ag/Alg, CNC/Alg, and CNC/Ag/Alg at 800 nm were valued at 92%, 67.37%, 48.27%, and 24.18%, respectively. The decrease in transmittance in all used films retained optical transparency, and no visual collections were detected (digital images of films presented in Figure 7C). The order of optical transparency (Table S2) of used films is as follows:

Alg > CNC/Alg > Ag/Alg > CNC/Ag/Alg

90.77 > 46.88 > 32.85 > 12.11

Although the Ag/Alg, CNC/Alg, and CNC/Ag/Alg composite films showed lower transparent results compared to neat Alg film which is good feature for packaging materials. The light transmittance at different wavelengths of Ag/Alg, CNC/Alg, and CNC/Ag/Alg bionanocomposite in the UV region was presented in Table S2. From Figure 7A, it was found that the CNC/Ag/Alg bionanocomposite films had more UV resistance power comparison to others used films. It might be due to uniform-homogenous dispersion of CNC and Ag nanoparticles in Alg matrix. The Hosseini group [71] and Arfat group [72] reported that UV light barrier property of biopolymer films can be improved by adding nanofiller. From the above results, it is clear that that CNC/Ag/Alg bionanocomposite films can be used as a UV barrier in food packaging materials.

### 3.9. Biodegradation of Films in Soil

**Figure 8:**
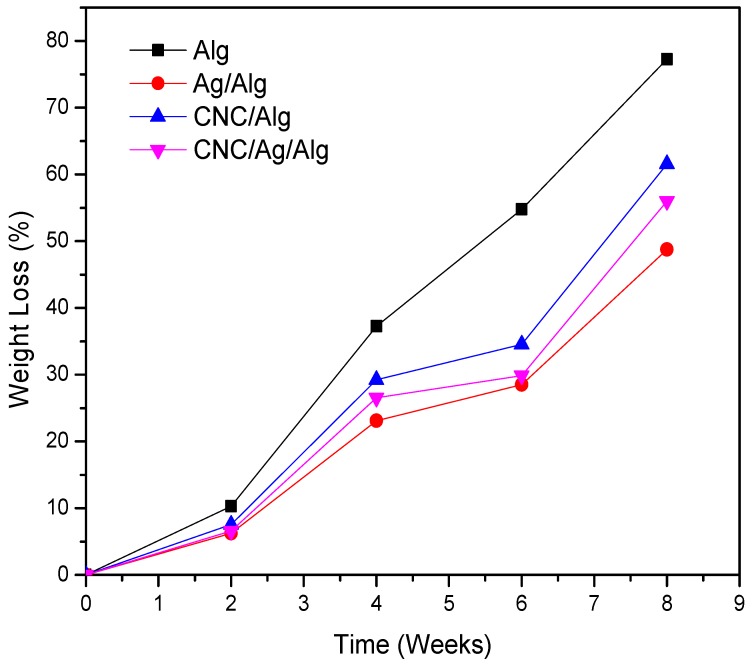
Biodegradation curves for Alg, Ag/Alg, CNC/Alg, and CNC/Ag/Alg films.

Biodegradability test is a highly important tool to recognize the environmental compatibility of materials. The biodegradability of food materials in the soil is already reported by many research group [73,74]. Specifically, the renewable biopolymer food packaging films showed an interesting biodegradable nature with soil. The biodegradation test of the prepared packaging films is assigned via soil burial degradation method for 8 weeks (Figure 8). The biodegradability results of Alg, CNC/Alg, Ag/Alg, and CNC/Ag/Alg films are presented in Figure 8. Evidently, the biodegradability of Alg films increased to 77.27% as the burial time increased in the soil for 8 weeks. The biodegradability rate of (5 wt.%) CNC/Alg bionanocomposite films was found to decrease in soil compared to Alg. It might be due to a strong interaction development between the matrix and the filler due to the homogeneous dispersion of CNC nanoparticles in the Alg matrix. Thus, the film degradation becomes complex due to difficulty in breaking the strong bonds between the Alg matrix and the CNC [30]. Meanwhile, Ag/Alg and CNC/Ag/Alg films showed a slight decrease in biodegradability behavior compared with Alg and CNC/Ag films due to the existence of metal (Ag).

## 4. Discussion

In this article, the needle-shaped CNC was prepared from CMC using acid hydrolysis and was used as reinforcement materials in bionanocomposites on the basis of Ag/Alg mixture. The solution blend technique was applied for fabricating CNC/Ag/Alg composite films. FTIR revealed that CNC and AgNPs interacted with Alg through hydrogen bonding, and good miscibility was developed among Alg, CNC, and Ag. The AgNPs-included composite films showed a specific plasmonic effect on the AgNPs with maximum light absorption at 491 nm and all the composite films, especially composed of AgNPs, showed the UV barrier property. The improved UV barrier property of the composite film can be used as a UV-screening food packaging film. Mechanical strength (determined by the TS), water barrier property, and thermal stability (determined by TGA analysis) of all the nanocomposite films increased significantly. The biodegradability of Alg and CNC/Alg films was found higher than other samples due to the absence of Ag metal. Based on the obtained results, the synthesized biodegradable nanocomposite films have the potential to be used as a food packaging material by using their barrier (water and UV) properties.

## Figures and Tables

**Table 1:** Values of thickness, film water solubility (FWS), moisture absorption (MA), water vapor permeation (WVP), Tensile Strength (TS) and Elongation at break (EB) (%) for Alg, Ag/Alg, CNC/Alg, and CNC/Ag/Alg films.

Sample Code	Thickness (μm)	FWS	MA (%)	WVP (×10^−11^gm^−1^s^−1^Pa^−1^)	TS (MPa)	EB (%)
Alg	10	99.20	18.87	6.53	25.60	17.10
Ag/Alg	11	89.39	12.33	4.20	40.30	13.20
CNC/Alg	11	61.29	12.22	5.40	35.50	14.30
CNC/Ag/Alg	12	56.36	13.24	4.60	39.40	14.70

**Table 2:** Opacity and color variables of Alg, CNC/Alg, Ag/Alg, and CNC/Ag/Alg films.

Sample Code	Opacity (Abs600/mm)	L	a	b	ΔE
Alg	1.37	93.68	–1.04	2.772	2.53
CNC/Ag	11.43	93.37	–0.1	4.77	4.22
Ag/Alg	20.84	32.95	9.33	14.02	65.02
CNC/Ag/Ag	37.70	36.92	29.95	28.10	71.77

## Supplementary Materials

The following are available online at https://www.mdpi.com/2079-4991/9/11/1523/s1, Table S1: Thermal stability of CMC, CNC, Alg, CNC/Alg, Ag/Alg and, CNC/Ag/Alg samples, Table S2: Light Transmittance and transparency of control and bionanocomposite films, Figure S1: Cross-sectional FESEM image of CNC/Ag/Alg (a & b), FESEM-EDS of CNC/Ag/Alg (c and d), and (e) EDX elemental mapping of CNC/Ag/Alg nanocomposite for the following elements: (f) C, (g) O, (h) Ag, and (i) Na, Figure S2: OM images of (a) CMC (b) CNC in dry powder form, (c) Alg (d) Ag/Alg (e) CNC/Alg (f) CNC/Ag/Alg in film form at Magnification 20×.
