# Supplementary material for: Fabrication of Cellulose Nanocrystal/Silver/Alginate Bionanocomposite Films with Enhanced Mechanical and Barrier Properties for Food Packaging Application
Source: Nanomaterials (Basel). 2019 Oct 25;9(11):1523. doi: 10.3390/nano9111523 (PMC6915576; doi:10.3390/nano9111523)
Supplement: Supplementary file 1 [file nanomaterials-09-01523-s001.pdf]

nanomaterials

MDPI

Supplementary materials

Fabrication of Cellulose Nanocrystal/Silver/Alginate Bionanocomposite Films with Enhanced Mechanical and Barrier Properties for Food Packaging Application

Mithilesh Yadav [1,*], Yu-Kuo Liu [1] and Fang-Chyou Chiu [1,2,*]

[1] Department of Chemical and Materials Engineering, Chang Gung University, Taoyuan 333, Taiwan ROC; ykliu@mail.cgu.edu.tw
[2] Department of General Dentistry, Chang Gung Memorial Hospital, Taoyuan 333, Taiwan ROC
* Correspondence: maxson@mail.cgu.edu.tw (F.-C.C.); dryadavin@gmail.com (M.Y.); Tel.: +88-69-5367-8628 (F.-C.C.); +88-69-0547-3797 (M.Y.)

Table S1. Thermal stability of CMC, CNC, Alg, CNC/Alg, Ag/Alg and, CNC/Ag/Alg samples.

Samples	S_1	S_2	S_3	A^*	K^*	IPDT(°C)
CMC	26876.3	519.79	4519.79	0.47152	1.16817	396.451
CNC	29117.8	863.75	5400.50	0.46672	1.06401	361.443
Alg	19988.3	14072.60	2839.20	0.50913	1.70404	601.934
CNC/Alg	18720.4	15256.80	329969.00	0.50732	1.81498	642.551
Ag/Alg	13901.5	24134.00	7150.70	0.58349	2.73607	1078.890
CNC/Ag/Alg	16721.1	17680.1	28652.7	0.54558	2.05735	766.312

Table S2. Light Transmittance and transparency of control and bionanocomposite films.

Samples	Light transmittance at different wavelength (%)							Transparency
	200nm	280nm	350nm	400nm	500nm	600nm	800nm	
Alg	1.55	75.05	86.22	89.87	91.28	91.59	92.00	90.77
Ag/Alg	0.08	5.56	13.57	13.26	7.62	26.09	67.37	32.85
CNC/Ag	0.15	35.28	43.47	45.83	47.18	47.80	48.27	46.88
CNC/Ag/Alg	0.06	1.85	4.57	5.02	2.68	8.67	24.18	12.11

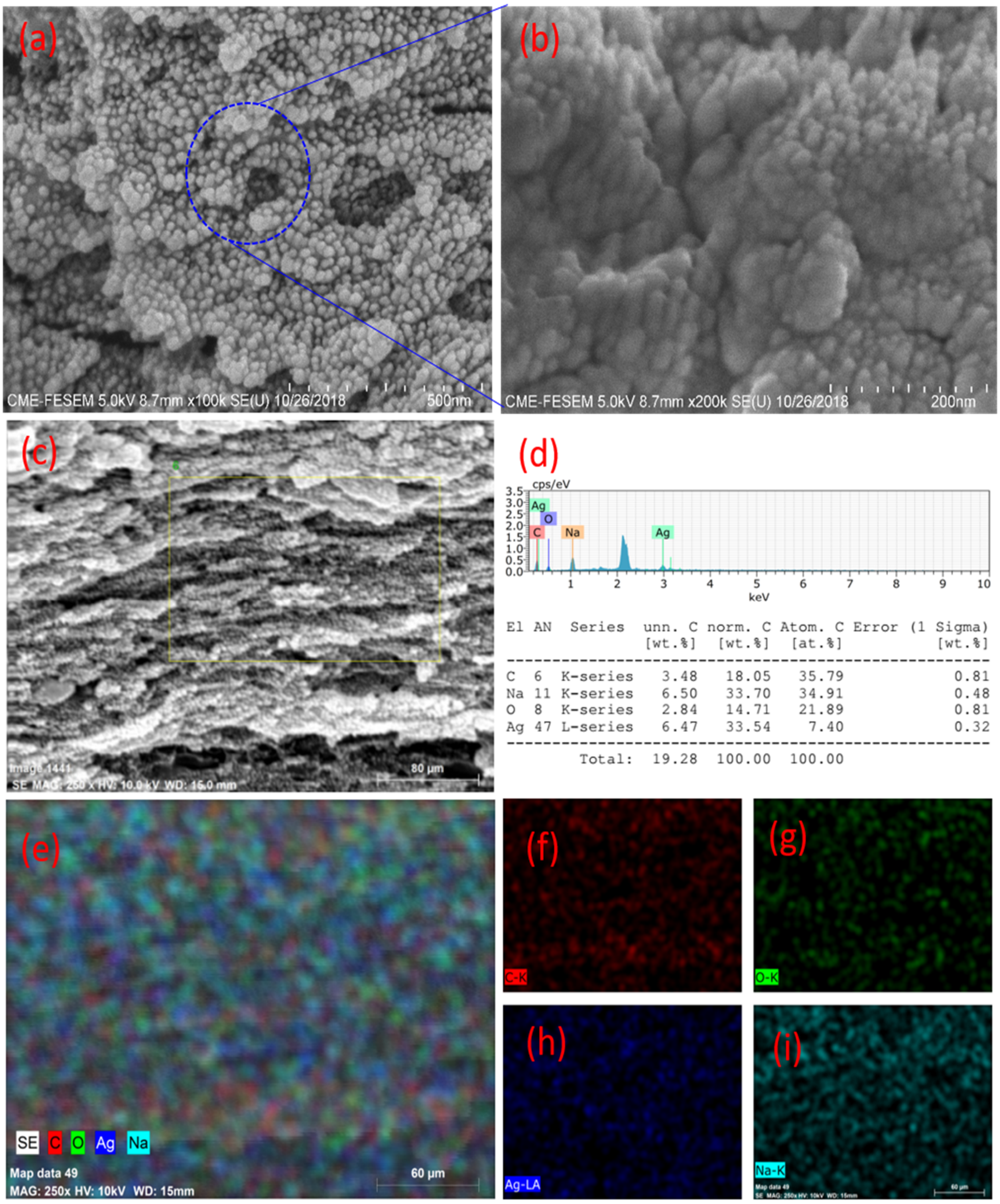

Figure S1. Cross-sectional FESEM image of CNC/Ag/Alg (a & b), FESEM-EDS of CNC/Ag/Alg (c and d), and (e) EDX elemental mapping of CNC/Ag/Alg nanocomposite for the following elements: (f) C, (g) O, (h) Ag, and (i) Na.

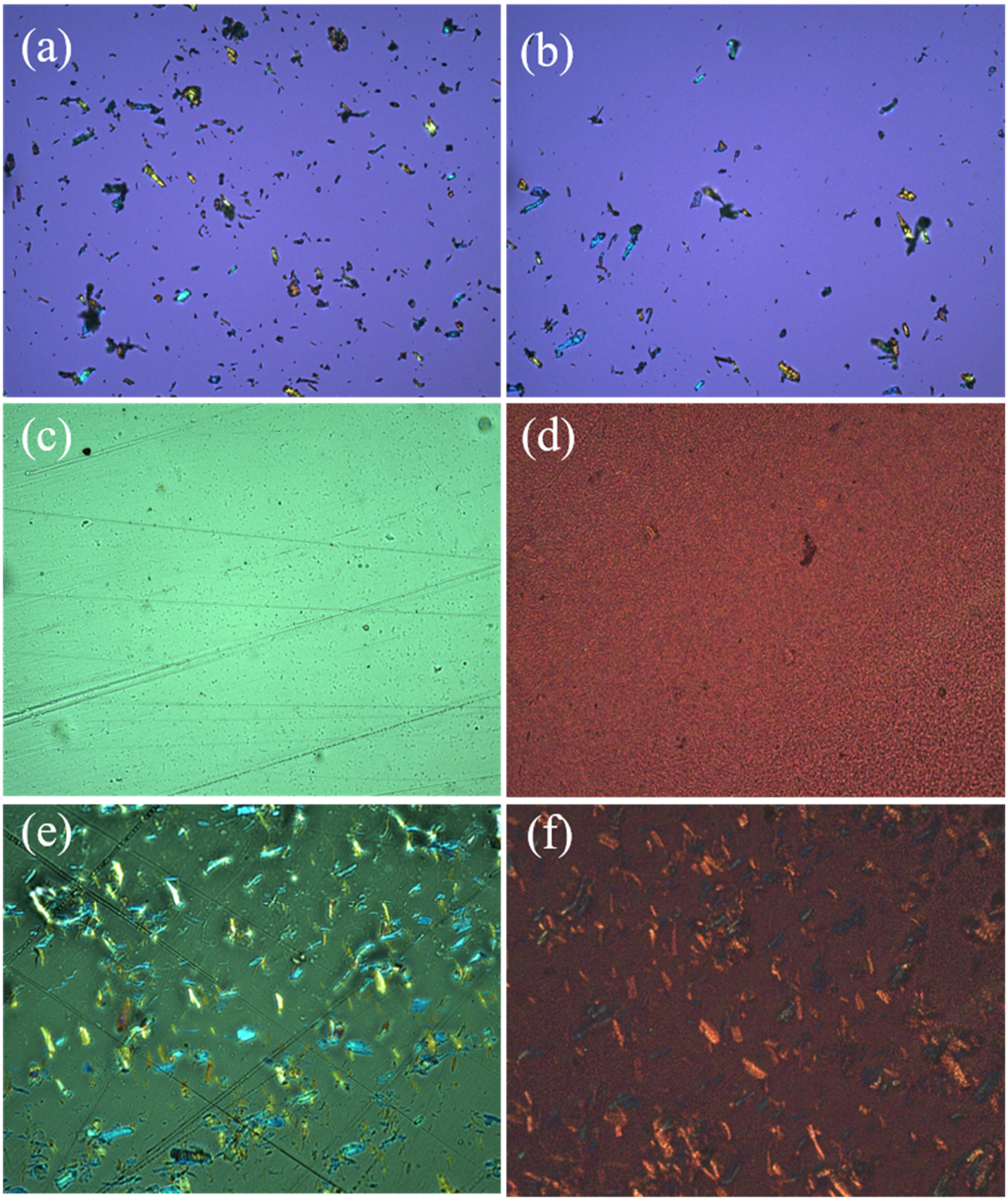

Figure S2. OM images of (a) CMC (b) CNC in dry powder form, (c) Alg (d) Ag/Alg (e) CNC/Alg (f) CNC/Ag/Alg in film form at Magnification 20×.
